# To Model or not to Model, That is no Longer the Question for Ecologists

**DOI:** 10.1007/s10021-016-0068-x

**Published:** 2017-03

**Authors:** Rupert Seidl

**Affiliations:** Institute of Silviculture, Department of Forest- and Soil Science, University of Natural Resources and Life Sciences (BOKU) Vienna, Peter Jordan Straße 82, 1190 Vienna, Austria

**Keywords:** ecosystem modeling, philosophy of science, ecological megatrends, education in ecology, computer simulation, model development

## Abstract

Here, I argue that we should abandon the division between “field ecologists” and “modelers,” and embrace modeling and empirical research as two powerful and often complementary approaches in the toolbox of 21st century ecologists, to be deployed alone or in combination depending on the task at hand. As empirical research has the longer tradition in ecology, and modeling is the more recent addition to the methodological arsenal, I provide both practical and theoretical reasons for integrating modeling more deeply into ecosystem research. Empirical research has epistemological priority over modeling; however, that is, for models to realize their full potential, and for modelers to wield this power wisely, empirical research is of fundamental importance. Combining both methodological approaches or forming “super ties” with colleagues using different methods are promising pathways to creatively exploit the methodological possibilities resulting from increasing computing power. To improve the proficiency of the growing group of model users and ensure future innovation in model development, we need to increase the modeling literacy among ecology students. However, an improved training in modeling must not curtail education in basic ecological principles and field methods, as these skills form the foundation for building and applying models in ecology.

## Introduction

In the not too distant past, deciding whether “to model or not to model” was a far-reaching decision for ecologists, firmly dividing the field into camps of “modelers” and “field ecologists.” Frequently, these labels went beyond being a succinct summary of the methodological approaches employed by someone, and came fraught with a suit of preconceptions about the mode of scientific inquiry of the respective other camp: “Field ecologists” would criticize “modelers” for choosing the easy way, evading the tedious weeks and months of data collection in the field, and simply “making up the data” on their computers in order to maximize their research output. In turn, “modelers” would call into question the proposition by “field ecologists” that another summer spent in the field would bring about *the* watershed event for answering a particular research question, let alone unearth new insights into how ecosystems function in general (that is, beyond the particular study system). In this not too distant past, you were initiated into the academic world a “modeler” or a “field ecologist,” often inheriting the affiliation to one or the other camp from your PhD advisor and lab (sometimes regardless of what approaches you actually chose to apply during and after your PhD).

Of course, the world is not (and never was) as black and white as this introductory paragraph suggested. As ecologists, we are well aware that clustering a highly diverse population of scientists into two camps will necessarily result in a considerable amount of unexplained variation. Furthermore, ecosystem ecology has quite a long track record of combining empirical and modeling approaches for ecological inquiry (for example, the International Biological Program, see Aronova and others (2010)). Here, I suggest that we go one step further and embrace modeling and empirical research as two powerful and often complementary approaches that should be in the toolbox of every 21st century ecologist, to be deployed alone or in combination, depending on the task at hand. As empirical research has the longer tradition, and modeling is the more recent addition to the methodological arsenal (despite also going back almost a century (Lauenroth and others 2003)), I will develop my argument from the perspective of adding modeling to the mix. The second reason for choosing this perspective is that it is (in my personal experience) much easier to convince ecologists of the need for more field research than it is to argue for more modeling in ecology. I will start with a brief summary of both practical and theoretical reasons for integrating modeling more deeply into ecosystem research. Yet, as I will elaborate in subsequent sections, for models to unfold their full potential, and for modelers to wield this power wisely, empirical research is of fundamental importance. The final section of the text will address the crucial question of implications for training 21st century (ecosystem) ecologists. Throughout the text, when using the term model, I primarily refer to dynamic and quantitative models, or simulation models, i.e., mathematical representations of ecosystems as a set of state and flow variables that dynamically interact and change over time.

## Practical Considerations for Integrating Modeling into Ecosystem Research

The practical motivation for making models an increasingly integral part of our methodological toolbox is that modeling is particularly well suited for addressing emerging new objectives and challenges in ecosystem ecology. Ecosystems research is rapidly increasing in complexity. One example is the growing realization that solely focusing on ecosystem processes might not suffice to understand the earth system, and that the influence of and interactions with humans require increasing consideration (Liu and others 2007). In other words: The advent of the Anthropocene (Steffen and others 2007) calls for a revision of our system boundaries when we study ecosystems and their functioning. Yet, a broadening of system boundaries necessarily increases complexity. Models can help us to cope with such increasing levels of complexity, in at least two ways: First, they allow us to consistently and quantitatively study the effect of complex interactions within a system. Keeping track of dynamic feedbacks and interactions is a main strength of models, and harnessing this strength can lead to important and sometimes unexpected discoveries of ecosystem dynamics (see for example, Yue and others 2016). Second, modeling can help us to identify which variables and interactions within a complex system are driving particular patterns of interest. A model is by definition a simplification of reality, and a lot can be learned about a system through the process of deliberate simplification that is at the core of model development. If, for instance, a complex ecological pattern is reproduced by a model consisting of only a small set of carefully selected variables and interactions, we might be one step closer to identifying key drivers of the observed pattern (for example, Wootton 2001). Vice versa, if we are not able to explain the observed system dynamics with a model that contains all the currently available process knowledge, modeling can aptly point to where our current limits in systems understanding lie, and what further research might be needed to advance the field. In this sense, even wrong models can be informative for pushing the frontier of ecosystem research.

A second mega-trend that bolsters the utility of models in ecology is the rapidly growing availability of data on a wide range of ecosystem characteristics. Earlier periods of ecosystem research were largely characterized by a scarcity of data, making data collection the key focus of many research programs, and limiting the ability to represent important ecosystem processes in models. However, we are in a rapid transition from an era of data limitation into an era of data wealth (Hampton and others 2013), and—as some might argue—even data overload. Contributing to this transition is the proliferation of remote sensing (for example, Kennedy and others 2014), large and coordinated research networks like Fluxnet and NEON (for example, Ershadi and others 2014), the use of citizen science (Jordan and others 2015), and a change in research culture towards making data accessible to the public (Hampton and others 2013). With regard to many research questions, the challenge is thus shifting from finding the data to test a hypothesized pattern, to finding and understanding patterns in the massive amounts of increasingly available data. Models are important tools in this regard, as they allow a consistent integration of different data streams towards a research question of interest (see for example, Thom and others 2016). Maybe even more importantly, as models are a quantitative rendering of our systems understanding, they are powerful tools for attributing patterns that emerge from big data to the underlying processes (for example, Piao and others 2015).

A third current mega-trend in ecology for which models are of integral importance is the increasing drive towards upscaling. The recent interest in macroecology (Heffernan and others 2014) is a direct response to emerging ecological challenges at the planetary scale, such as climate change and the currently ongoing global mass extinction of species (Steffen and others 2015). Also with regard to temporal scales, an extended perspective has come into focus recently, with the separation of ecological and evolutionary time scales beginning to blur (Schoener 2011). Because they allow a consistent extrapolation of processes from the level of their understanding (for example, carbon balance of plant organs) to the level of interest for decision makers (for example, the global carbon taken up by vegetation in the context of mitigating climate change), and because they enable the investigation of long-term trajectories in a resource-efficient manner, models are prime tools in the effort to scale up in space and time (Seidl and others 2013).

## Theoretical Considerations for Integrating Modeling into Ecosystem Research

The imperative towards a deeper integration of modeling not only originates from its practical uses in the context of the emerging new questions and trends in ecosystem research, but can also be substantiated based on theoretical grounds. From a philosophy of science perspective, recent decades were characterized by a slowing of theory development, while an avalanche of novel applications of existing theories emerged. The latter has been strongly aided by modeling, which is why the 21st century is already termed the age of computer simulation (Winsberg 2010).

A philosophy of science of computer simulation is only slowly emerging, yet it has been argued that simulation is an entirely new mode of scientific activity, one that lies between theory and empirical research. The epistemology of simulation is very much an empirical epistemology and not merely a mathematical or logical one. Both empirical science and simulation work with proxies for the actual study system of interest. Empirical research on the effect of diversity on ecosystem functioning, for instance, is frequently carried out in common garden experiments (Verheyen and others 2016), assuming that the relationships found in such a model system also apply to the real world. Modeling studies investigating similar questions are able to address many of the complexities that are neglected in empirical model systems (for example, long-term compositional changes in response to competitive interactions between species; the effect of low probability—high impact events such as disturbance (Silva Pedro and others 2016)). Yet, they in turn operate under the assumption that findings deduced from the mathematical approximation of the ecosystem in the computer apply to the real-world system.

Both empirical research and simulation build on assumptions of representation. The difference is that in empirical research the object (for example, a field of saplings in a common garden) bears a deep, material similarity with the intended target (for example, a forest ecosystem), whereas in simulation the object (the code representing the ecosystem in the computer) bears an abstract, formal similarity to its intended target (Guala 2002). In this way, modeling shifts the emphasis away from objects and rather focuses on configurations of processes (Ulanowicz 2009). Which of the two methods of inference is more appropriate thus strongly depends on whether material or formal similarity is of greater relevance in the context of the research question at hand. Yet, empirical research has epistemological priority over modeling (Winsberg 2010). For both approaches, we need to know something to learn something, but the prior knowledge needed for modeling is considerably greater than that required for conducting empirical research. Modeling necessarily relies on prior experiments and observations to build and evaluate models, which is why modeling should be conducted jointly with empirical research.

In addition to empirically collected data, the computational representation of the system in a simulation model is based on our theory of system dynamics. And although models are guided by theory, they are not necessarily determined by it. In other words, simulations can produce novel results that are not implicitly contained in the theory that guided their development (Winsberg 2010). In complex systems, for instance, the typical behavior of individual entities can often be described mathematically based on theory, given a set of initial conditions and boundaries. The typical long-term behavior of such systems, however, is an emergent property of the interactions between those entities. The singularity of the entities interacting in biological systems, in combination with the heterogeneous template provided by the natural environment, can lead to “combinatorics and heterogeneity overwhelming law” (Ulanowicz 2009) in living systems. This complexity can only be studied through patient long-term observation—or computer simulation (Bedau and Humphreys 2008). Simulations allow us to extend reductionism (that is, knowledge on isolated processes derived devoid of context) into new territory by including the effect of interactions and investigating the effect of novel contexts and conditions. Models are thus not mere dynamic renderings of theoretically derived equations, but can be seen as rich, physical constructs that mediate between our theories and the world (Winsberg 2010).

## Balancing Empirical and Model-Based Research

Considering the ecological challenges ahead (Steffen and others 2015), I maintain that we need to make use of the entire arsenal of approaches that is available to us and integrate modeling more strongly into ecosystem research. From this proposition, however, follows the question of the optimal level of such integration: Should every single paper in a journal such as Ecosystems henceforth include a modeling component? Should we design PhD curricula in ecosystem ecology so that at least one chapter of every PhD will make use of modeling? Is it at the level of project cycles or career stages that such integration is best achieved, with researchers switching from empirical work to modeling in three- to ten-year cycles? Or is the level of the individual researcher the wrong scale entirely, and the best course of actions is to ensure that dedicated modeling experts make efficient use of the approach within the population of researchers within the field?

There is no single correct answer to this question, as is the case with so many questions of scale. Yet, I would caution against the endpoints of the just described scale spectrum, and suggest that it is at the intermediate levels, that is, an integration at the level of individual PhDs or three- to ten-year career stages, where we can gain the most from embracing modeling as a methodological pillar of ecosystem research. That a strong integration of different methodological approaches at the level of individual papers can be problematic will be obvious to most, as it decreases the methods-to-results ratio. Page space in scientific journals is increasingly scarce, while at the same time, methodological approaches are becoming more and more complex, intensifying the struggle to describe methods in a way that readers are able to understand and reproduce them. Pushing for a stronger integration of methodological approaches at the level of individual articles might thus further aggravate this problem. However, that is, not to say that a combination of the inferential potential of empirical and modeling approaches in individual articles cannot make for particularly insightful studies (see for example, Seidl and others 2012).

At the other end of the spectrum lies the world that I have sketched in the introductory section of this text, in which the balance between empirical and modeling approaches is achieved at the population level. Such a strict division has strong disadvantages, as it discourages cross-pollination between methods, and underutilizes the power of multi-method inference. Furthermore, increasingly narrow methodological niches and specialization make the communication of ideas and results difficult, stymie collaboration within the field, and discourage the flexible and interdisciplinary approaches that are needed to address emerging global challenges. The sweet spot of integrating modeling into our toolboxes as ecosystem ecologists, the Medawar zone yielding the highest payoff (Loehle 1990), lies between these poles. Where exactly will vary from individual to individual, with some preferring to work with different approaches simultaneously while others go about it serially and master one method first, adopting another one later. A third promising option is close and long-standing collaborations between people using different methodological approaches. Such “super ties” have recently been demonstrated to be highly beneficial for scientific productivity (Petersen 2015), and facilitate mutual understanding and a creative utilization of a wide spectrum of tools and approaches.

## Increasing Modeling Literacy in Future Generations of Ecologists

The power of modeling as a methodological approach of ecosystems research is much more widely recognized than exercised, a fact that has previously been attributed to inadequate training in modeling (Lauenroth and others 2003). Consequently, increasing the modeling literacy among future ecologists is a key factor towards a better utilization of modeling and its potential as methodological approach. I suggest that a basic understanding of modeling, together with skills regarding data analysis and programming, should be included in any ecology curriculum. Some aspects of ecology might even be easier to teach using models, such as the potential effect of feedbacks on ecosystem dynamics (Yue and others 2016). Understanding terms and concepts such as drivers versus parameters, state versus flux variables, and the difference between averaging the input versus output of a nonlinear equation (Jensen’s inequality, (Ruel and Ayres 1999)) will not only enable students to more easily adopt a modeling approach to their future questions, but will also make them better ecologists altogether. Also hands-on exposure to modeling is important, for example, tinkering with model formulations, inputs, parameters, and outputs, as this will strongly increase the intuitive understanding of basic principles of modeling in students.

Training more adept model users is urgently needed as the proliferation of modeling progresses. Improvements in software design, standardization of interfaces, and convenient availability through the worldwide web have made it easier to access and apply models in recent years. Lower technical barriers will likely further increase the number of model users in the future, in analogy with how (R Development Core Team 2016) and the increasing availability of specialized statistical libraries have proliferated the use of complex statistical analyses in ecology. However, the model user is ultimately more important than the model itself for the outcome of an analysis, or “A smart analyst with a simple model can work wonders. A powerful model in the hands of an inept analyst is like a toddler with a machine gun” (Nelson 2003). It is thus increasingly important for future ecologists to understand the inherent differences between empirical and modeled data (for example, regarding variability or sample size), to know about the strengths and limitations of particular modeling approaches, and consider them carefully in their applications.

Although every ecology student should thus attain at least a basic level of proficiency as model user, some will be enticed by the power and possibilities of learning about real ecosystems from emulating them in silico. They will be curious about what is “under the hood” of a model they have been tinkering with, which arguably provides the best segue into becoming more deeply involved in model development. From this smaller cohort of students, the next generation of model developers will be recruited, that is, they will be shaping the future of ecosystem modeling. But do we really need new model development, are there not enough models out there already, one might ask? I wouldd argue that, whereas there is no harm in having more (and more specialized) tools in our toolbox, a small or decreasing choice of models (resulting from a decreasing number of people engaging in the daunting task of developing new models, *inter alia* resulting from a decreasing propensity of funding agencies to support such ventures) holds considerable risks for the community. First, research questions change faster than the available methodological approaches do. This increases the likelihood that models will be applied to questions outside of the domain that they have been developed for initially. Continuous model development is thus important not only to integrate newly available data and computational approaches, but also to ensure that we are using the right tools to answer emerging new questions. Second, a narrowing diversity in model formulations has the potential to induce a false sense of certainty in their collective projections. Multi-model ensembles are frequently used to quantify model-related uncertainties (for example, Warszawski and others 2013). Yet, if the large majority of available models rely on the same underlying process formulations (for example, with regard to how photosynthesis, respiration, transpiration, or disturbance is modeled), the degree of agreement within the ensemble is a poor indicator of process uncertainty. We need continued innovation in model development, which also means we need to spark the interest for modeling in future generations of ecologists, and ensure that choosing ecosystem modeling is a promising and worthwhile career path for young ecologists.

## The Importance of Exposure to the Study System

At this point in the text, the plea for more and better education in ecological modeling probably comes as no surprise. An interesting question that remains in this context, however, is: What should an intensified modeling training be substituted for? Because the materials and approaches that can be covered in ecology programs are necessarily limited, and there is an eternal tug-of-war regarding credits and courses within university departments, what trade-offs should we be accepting in this regard? Again, there is no single answer to this question, yet, I would strongly caution against trading off education in basic ecological understanding and field methods for an improved education in modeling. In analogy with the epistemological priority of empirical approaches described above, these skills are the foundation on which any ecosystem modeling is built, and curtailing them would ultimately counteract the aim to increase modeling literacy. If, for instance, the basic processes of an ecosystem are not understood, or if available empirical data are misinterpreted because of a lack of understanding of the underlying field methods, any modeling attempts will be doomed from the start. I have argued before that understanding basic principles of modeling can help people in becoming better ecologists; I equally vehemently maintain that extensive exposure to the field makes for a better model(er). Among the many benefits, time spent in the field can help modelers to better understand the variability inherent in ecosystems, and counteract an overly strong focus on the central tendency in modeling. As it is the outliers that often spark new insights, a stronger consideration of variability by modelers—ushered in by more exposure to the field—would likely benefit model development and interpretation. Furthermore, differences between observed and simulated trajectories are often reflexively attributed to possible misspecifications of the model. Yet, they could also be interpreted as the model pointing to alternative pathways of system dynamics. Whether unexpected model behavior results from an ill-defined model or suggests the possibility of a hitherto unrecognized system trajectory can only be discerned by searching for evidence of the dynamics suggested by the model in the field. Modeling thus opens up new perspectives for empirical ecosystem research.

In summary, I propose that a deep integration of modeling into the arsenal of approaches applied in ecology should be the new normal, rendering the question of whether “to model or not to model” obsolete. Such integration will not only increase the inferential power in the context of current challenges, but will also obliterate any lingering remnants of the divide between “field ecologists” and “modelers.” Applied in the context of teaching, an integrated training in both empirical and modeling approaches will enable future generations of ecologists to creatively choose from a diverse methodological toolbox in addressing the challenges of the 21st century.
